# Efficacy and safety of valbenazine in Japanese patients with tardive dyskinesia: A multicenter, randomized, double‐blind, placebo‐controlled study (J‐KINECT)

**DOI:** 10.1111/pcn.13455

**Published:** 2022-09-17

**Authors:** Jun Horiguchi, Koichiro Watanabe, Kazuoki Kondo, Atsushi Iwatake, Hajime Sakamoto, Yutaka Susuta, Hideaki Masui, Yumi Watanabe

**Affiliations:** ^1^ Department of Psychiatry Shimane University Faculty of Medicine Izumo Shimane Japan; ^2^ Department of Neuropsychiatry Kyorin University School of Medicine Mitaka Tokyo Japan; ^3^ Ikuyaku, Integrated Value Development Division Mitsubishi Tanabe Pharma Corporation Osaka Japan

**Keywords:** abnormal involuntary movement scale, randomized controlled trial, tardive dyskinesia, valbenazine, vesicular monoamine transporter 2

## Abstract

**Aim:**

Valbenazine is approved in the US for treatment of tardive dyskinesia (TD); however, efficacy/safety data in Asian populations are lacking. We assessed the efficacy/safety of valbenazine in Japanese patients.

**Methods:**

This phase II/III, multicenter, randomized, double‐blind, placebo‐controlled study (NCT03176771) included adult psychiatric patients with TD, who were randomly allocated to receive placebo or valbenazine (once‐daily 40‐ or 80‐mg) for a 6‐week, double‐blind period, after which the placebo group was switched to valbenazine for a 42‐week extension. The primary endpoint was change from baseline in Abnormal Involuntary Movement Scale (AIMS) total score at Week 6; clinical global impression of improvement of TD (CGI‐TD) was also assessed.

**Results:**

Of 256 patients, 86, 85, and 85 were allocated to the 40‐mg valbenazine, 80‐mg valbenazine, and placebo groups, respectively. Least‐squares mean (95% confidence interval) change from baseline in AIMS score at Week 6 was −2.3 (−3.0 to −1.7) in the valbenazine 40‐mg group, −3.7 (−4.4 to −3.0) in the 80‐mg group, and −0.1 (−0.8 to 0.5) in the placebo group; both treatment groups showed statistically significant improvements *vs.* placebo. Patients switched to valbenazine at Week 6 showed similar improvements in AIMS scores, which were maintained to Week 48. Improvements in CGI‐TD scores were observed for both treatment groups *vs.* placebo. Incidence of adverse events was highest in the 80‐mg group; common events included nasopharyngitis, somnolence, schizophrenia worsening, hypersalivation, insomnia, and tremor.

**Conclusion:**

The efficacy/safety profile of valbenazine was similar to that of previous clinical trials, supporting its use for TD treatment in Japanese patients.

Tardive dyskinesia (TD) is a neurological condition associated with prolonged exposure to dopamine receptor‐blocking agents such as antipsychotic medications.^1^ Although first‐generation antipsychotics (FGAs) are more commonly associated with TD than the more recently developed second‐generation antipsychotics (SGAs), TD remains a troublesome and potentially serious clinical consideration related to the use of both FGAs and SGAs.^2^

The primary symptoms of TD are involuntary movements of the orofacial region, including tongue, lips, and jaw, as well as the extremities and trunk.^3^ Even mild symptoms cause a decline in patients' quality of life, to some extent, depending on the individual's living environment, and moderate to severe symptoms cause considerable physical disability.^4^ In extreme cases, the condition may become life‐threatening, such as when the esophageal or respiratory muscles become involved and patients may have difficulty swallowing or breathing.^5^, ^6^, ^7^

Although various treatments have demonstrated efficacy in reducing symptoms, there is no cure for TD; therefore, it remains an important concern for patients who require antipsychotic medication to effectively control psychiatric symptoms. With the advent of SGAs and the belief that they are less likely to induce TD, their clinical use has expanded to include off‐label indications for psychiatric disorders other than schizophrenia, such as major depressive disorder and anxiety disorders.^8^, ^9^, ^10^ Therefore, the incidence of TD may continue to increase, despite the waning use of FGAs.

Small‐scale studies investigating possible treatments for TD have reported mixed results.^11^ Among the most promising new therapies for TD are the vesicular monoamine transporter 2 (VMAT2) inhibitors, a new drug class including valbenazine and deutetrabenazine.^12^, ^13^, ^14^, ^15^, ^16^, ^17^ The safety and efficacy of VMAT2 inhibitors for treatment of TD has been demonstrated in numerous overseas clinical trials, and both valbenazine and deutetrabenazine are currently approved in the US for this indication.^18^ However, pre‐approval clinical trials conducted overseas for VMAT2 inhibitors recruited very few Asian patients: over 90% of the population of the KINECT‐3 valbenazine trial was non‐Asian^12^ and in the AIM‐TD deutetrabenazine study, the majority of patients were white.^13^ Until very recently, there were no VMAT2 inhibitors approved in Japan for the treatment of TD, and our study is the first to study valbenazine specifically in an Asian population. The present study was therefore conducted to investigate the efficacy and safety of once‐daily 40‐ or 80‐mg oral valbenazine for the treatment of TD in Japanese psychiatric patients.

## Methods

### Study design

**Fig. 1:**
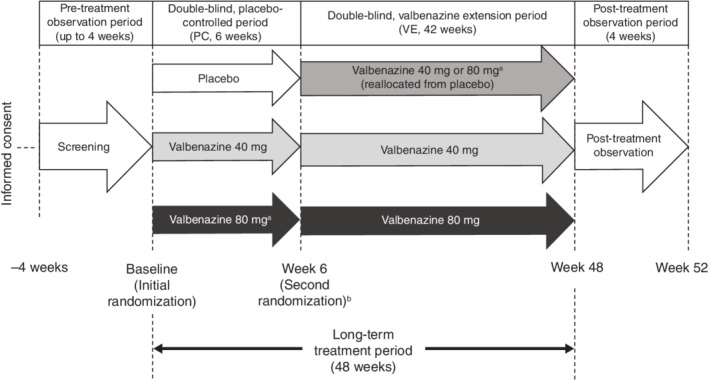
Study design. ^a^Patients allocated to the 80‐mg dose initiated valbenazine at a daily dose of 40 mg for 7 days before uptitrating to 80 mg. ^b^For the extension period, patients who received 40‐ or 80‐mg valbenazine during the placebo‐controlled period continued at the same dose. Patients who received placebo during the placebo‐controlled period were randomly reallocated to either 40‐ or 80‐mg valbenazine for the extension period. PC, placebo‐controlled; VE, valbenazine extension.

This multicenter, phase II/III, randomized, double‐blind, placebo‐controlled, parallel‐group, fixed‐dose study was conducted across 100 sites in Japan, including national hospitals and outpatient private clinics. The study was initiated on 21 June 2017 and completed on 29 September 2020. The trial design is illustrated in Fig. 1. The study period consisted of a pre‐treatment observation period of up to 4 weeks (screening period); a double‐blind, placebo‐controlled (PC) period of 6 weeks; a double‐blind 42‐week valbenazine extension (VE) period; and finally a 4‐week post‐treatment observation period. This study was performed in accordance with the ethical principles of the Declaration of Helsinki, and in compliance with the “Act on Securing Quality, Efficacy and Safety of Pharmaceuticals, Medical Devices, Regenerative and Cellular Therapy Products, Gene Therapy Products, and Cosmetics,” “Good Clinical Practices (GCP),” and the protocol. The protocol was approved by the relevant institutional review boards for each study center. All patients provided written informed consent prior to initiating the screening period. The study was registered at ClinicalTrials.gov under the identifier NCT03176771.

The inclusion criteria were as follows: men and women; aged 20–85 years at the time of informed consent; diagnosed with schizophrenia, schizoaffective disorder, bipolar disorder, or major depressive disorder according to the fifth edition of the Diagnostic and Statistical Manual of Mental Disorders (DSM‐5) at least 3 months prior to informed consent; diagnosed with TD (DSM‐5 code 333.85) that was judged to be moderate to severe based on evaluation using the Abnormal Involuntary Movement Scale (AIMS)^19^ item 8; with stable maintenance treatment for their primary psychiatric disorder for at least 30 days (14 days for benzodiazepines) before initiation of the screening period; and a body mass index of 17.0–<35.0 kg/m^2^. The major exclusion criteria were active, clinically significant, unstable cerebrovascular, hepatic, renal, endocrine, cardiovascular, gastrointestinal, respiratory, or metabolic disease; presence of other prominent comorbid movement disorder (e.g., dystonia, akathisia, parkinsonism) that would interfere with assessment of TD; diagnosis of major neurocognitive disorder according to the DSM‐5; presence of substance abuse disorder; high risk of suicidal or self‐injurious behavior as judged by the investigator or suicide attempt or suicidal ideation corresponding to item 4 or 5 of the Columbia Suicide Severity Rating Scale assessment at baseline (within 3 months before evaluation); and unwillingness to use appropriate contraception from the time of informed consent until 28 days after study discontinuation. A full listing of all exclusion criteria, along with relevant protocol amendments, is provided in Appendix S1.

At the time of informed consent, patients were instructed that they would receive either placebo or valbenazine (40‐ or 80‐mg) for the initial 6 weeks, then 40‐ or 80‐mg valbenazine for the 42‐week VE period. After completion of the screening period, patients were randomly allocated to three groups (40‐mg valbenazine, 80‐mg valbenazine, or placebo). Patients received two capsules to be taken orally, once daily (in the morning) for 6 weeks, at which point the primary efficacy assessment was conducted. Thereafter, the placebo group was reallocated to receive either 40‐ or 80‐mg valbenazine. Any patient allocated to 80‐mg valbenazine first initiated valbenazine at a daily dose of 40 mg for 7 days before dose escalation to 80 mg. Dose reduction was not permitted during the PC period. In the case of dose reduction because of treatment‐emergent adverse events (TEAEs), patients receiving 80 mg could undergo a reduction to 40 mg, but for patients receiving 40 mg, the same dose was continued in a blinded manner (only placebo capsules were discontinued), and the treatment was discontinued entirely if there was no improvement in symptoms. To assess treatment compliance, patients were instructed to bring all investigational product supplies to each visit (including empty drug packages) and the investigator recorded use in the case report form.

#### Randomization and blinding

Our study used block randomization stratified by underlying disease (schizophrenia or schizoaffective disorder, bipolar disorder, or major depressive disorder). Patients were initially randomly allocated in a 1:1:1 ratio to receive 40‐ or 80‐mg valbenazine or placebo. At the end of the 6‐week PC period, patients who received placebo were reallocated in a 1:1 ratio to receive 40‐ or 80‐mg valbenazine; patients who received valbenazine during the initial 6‐week period continued at the same dose. The randomization key code table was prepared by BellSystem24, Inc. (Tokyo, Japan), and the sponsor provided the investigational product with assigned drug numbers to the study investigators. BellSystem24, Inc. retained the material list, the randomization key code table, and the patient number comparison table data until unblinding, and the sponsor's chemistry, manufacturing, and quality control department retained the material list until unblinding. Study investigators were blinded to the treatment using drug numbers confirmed through a web‐based registration system. Patients received two visually indistinguishable capsules of the investigational product: the 80‐mg group received two 40‐mg capsules of valbenazine, the 40‐mg group received one 40‐mg capsule of valbenazine and one placebo capsule, and the placebo group received two placebo capsules.

### Outcome measurements

The primary endpoint was the change from baseline in AIMS total score^19^, ^20^ as determined by central assessment at Week 6. Secondary efficacy endpoints included the percentage of patients with ≥50% improvement from baseline in AIMS total score; clinical global impression of improvement of TD (CGI‐TD) score^21^ at Week 6; and change from baseline in AIMS total score as determined by site assessment at Week 6. AIMS scores were also assessed at Weeks 2, 4, 16, 32, 48, and 52, and CGI‐TD scores were also assessed at Weeks 48 and 52.

The AIMS is a 12‐item scale used by physicians to assess the severity of TD symptoms. Items are scored on a scale ranging from 0 (“none”) to 4 (“severe”), and the total score includes items 1 through 7, which assess the severity of abnormal movement in the facial regions and throughout the body. Investigators (or subinvestigators) video recorded patients' movements and sent the recordings to the AIMS central assessment facility. AIMS central assessment was performed in a blinded manner by two neurologists skilled in the diagnosis and assessment of TD, after the evaluation time points of the video recordings had been randomized to ensure consistency in their assessments. The two raters were required to reach a consensus on the assigned score for each item. Site assessments were performed by individual investigators (or subinvestigators) who had undergone the necessary training and had been appropriately certified by CNS Ratings, LLC. The CGI‐TD is a clinician's assessment of change in TD symptoms, on a scale ranging from 1 (“very much improved”) to 7 (“very much worse”).

Throughout the study, patients were monitored for TEAEs and treatment‐related (possibly or definitely related to the study drug as assessed by the investigator) TEAEs (related TEAEs), which were coded using the Japanese version of the Medical Dictionary for Regulatory Activities version 23.0, and tabulated by System Organ Class and Preferred Term. Blood and urine sampling for clinical laboratory tests was performed at screening; baseline; Weeks 2, 4, 6, 8, 12, 16, 20, 24, 28, 32, 36, 40, 44, and 48; and at the end of the post‐treatment observation period. Vital signs and 12‐lead electrocardiograms were also performed and results were recorded in the case report forms. Patients' underlying psychiatric disease was assessed and compared with baseline scores of the following measures: the Japanese version of Calgary Depression Scale for Schizophrenia (JCDSS),^22^ the Japanese version of Montgomery–Asberg Depression Rating Scale (MADRS‐J),^23^ the positive and negative symptom scale (PANSS),^24^ and the Young Mania Rating Scale (YMRS).^25^

### Statistical analyses

As the pharmacokinetic profile of valbenazine is similar for Japanese and non‐Japanese patients, and no clinical studies of valbenazine have been conducted specifically in Japanese psychiatric patients, we based the sample size calculation on the results of an overseas phase III study.^12^, ^17^ The sample size required to obtain a significant difference in the AIMS total score at Week 6 in both 40‐ and 80‐mg groups *vs*. placebo with a power of 0.8 (2‐sided *t*‐test with α = 0.05) was 216 patients. Assuming a 10% dropout rate, we aimed to recruit at least 240 patients (80 per group).

The safety analysis set included all patients who received at least one dose of the study drug and had available post‐baseline safety data. Safety was assessed separately for the PC period, the long‐term treatment period (which included both the PC and VE periods), and post‐treatment periods. The efficacy analysis was conducted in the intention‐to‐treat set, and included all patients in the safety set who had available baseline and at least one post‐baseline AIMS total score (by central assessment). For the primary endpoint, each treatment group was compared with placebo using a mixed‐effect model for repeated measures (MMRM) with the group, underlying disease, and evaluation time point (Weeks 2, 4, and 6) as fixed effects, baseline as a covariate, and group × evaluation time point and baseline × evaluation time point as interactions. The variance–covariance matrix of within‐subject scores was unstructured, and the degree of freedom was calculated by the method of Kenward and Roger.^26^ Missing values were not imputed.

Descriptive statistics were used to report changes from baseline at the end of the post‐treatment observation period. The number and incidence of TEAEs were tabulated at each evaluation period. Statistical tests were two‐sided with a 5% level of significance. The statistical analyses for secondary endpoints are described in Appendix S1. The statistical analysis was conducted using SAS software v9.4 (SAS Institute Inc., Cary, NC, USA).

## Results

**Fig. 2:**
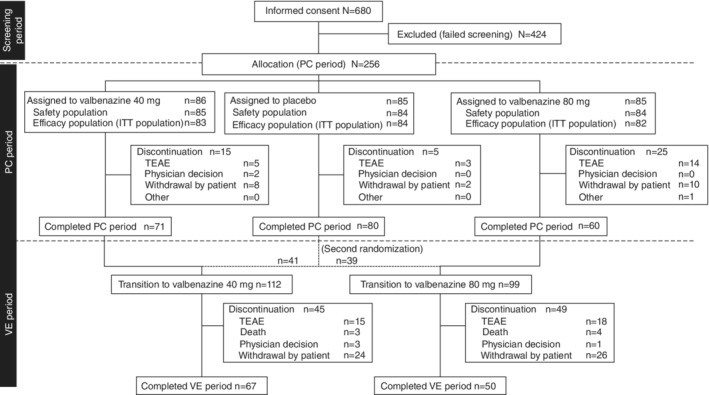
Patient disposition. TEAE, treatment‐emergent adverse event; ITT, intention‐to‐treat; PC, placebo‐controlled; VE, valbenazine extension.

Of 680 patients who provided informed consent, 86, 85, and 85 met all inclusion criteria and were randomly allocated to the valbenazine 40‐mg, valbenazine 80‐mg, and placebo groups, respectively (Fig. 2). The primary reasons for exclusion at the screening stage were TD severity not meeting the inclusion criteria or presence of significant involuntary movements other than TD. A total of 211 patients completed the 6‐week PC period and transitioned to the VE period. The most common reasons for discontinuation among the 45 patients who discontinued the study during the PC period were TEAEs and patient decision to withdraw. A further 94 patients discontinued during the VE period, also primarily owing to TEAEs and patient decision.

The patient background characteristics are shown in Table 1. For the valbenazine group, the mean ± standard deviation (SD) overall age was 58.2 ± 13.8 years, 53.3% of patients were men, the mean age at diagnosis of TD was 54.5 ± 14.7 years, and 73.9% of patients were using at least one antipsychotic medication at baseline. Most patients receiving valbenazine (64.8%) had a primary diagnosis of schizophrenia/schizoaffective disorder; 35.2% were diagnosed with bipolar or depressive disorder. The mean baseline AIMS dyskinesia total score was 7.6 ± 4.1. There were no notable between‐group differences in baseline demographics or characteristics.

**Table 1:** Baseline patient characteristics (intention‐to‐treat analysis set)

		Valbenazine		
	Placebo (*n* = 84)	40 mg (*n* = 83)	80 mg (*n* = 82)	Total (*N* = 165)
Age, years	60.0 ± 13.4	58.5 ± 14.0	57.9 ± 13.6	58.2 ± 13.8
Sex, male	36 (42.9)	38 (45.8)	50 (61.0)	88 (53.3)
Body mass index, kg/m^2^	23.75 ± 3.82	23.40 ± 4.00	23.90 ± 4.21	23.65 ± 4.10
Age at diagnosis of TD, years	57.0 ± 12.8	55.0 ± 14.4	54.0 ± 15.0	54.5 ± 14.7
Duration of TD, years				
<2	48 (57.1)	35 (42.2)	33 (40.2)	68 (41.2)
≥2	36 (42.9)	48 (57.8)	49 (59.8)	97 (58.8)
Disease category				
Schizophrenia/schizoaffective disorder	53 (63.1)	54 (65.1)	53 (64.6)	107 (64.8)
Age at diagnosis, years	42.4 ± 16.4	40.5 ± 15.2	36.8 ± 15.8	38.7 ± 15.5
Bipolar disorder/Major depressive disorder	31 (36.9)	29 (34.9)	29 (35.4)	58 (35.2)
Age at diagnosis, years	46.4 ± 13.9	45.4 ± 15.3	46.8 ± 14.5	46.1 ± 14.8
Use of antipsychotic medication, yes	67 (79.8)	59 (71.1)	63 (76.8)	122 (73.9)
1 medication	49 (58.3)	37 (44.6)	42 (51.2)	79 (47.9)
2 medications	17 (20.2)	16 (19.3)	21 (25.6)	37 (22.4)
≥3 medications	1 (1.2)	6 (7.2)	0 (0.0)	6 (3.6)
Use of SGAs only	57 (67.9)	47 (56.6)	48 (58.5)	95 (57.6)
Use of FGAs only or concomitant FGAs and SGAs	10 (11.9)	12 (14.5)	15 (18.3)	27 (16.4)
Chlorpromazine equivalent				
<600 mg	51 (60.7)	45 (54.2)	43 (52.4)	88 (53.3)
≥600 mg	16 (19.0)	14 (16.9)	20 (24.4)	34 (20.6)
Use of anticholinergics, yes	33 (39.3)	36 (43.4)	37 (45.1)	73 (44.2)
History of suicidal ideation and attempted suicide, yes	32 (38.1)	32 (38.6)	37 (45.1)	69 (41.8)
History of suicidality within 3 months prior to screening, yes	4 (4.8)	8 (9.6)	6 (7.3)	14 (8.5)
AIMS dyskinesia total score (central assessment)	8.0 ± 4.2	7.7 ± 3.8	7.4 ± 4.3	7.6 ± 4.1

Data are *n* (%) or mean ± standard deviation.

AIMS, abnormal involuntary movement scale; FGAs, first‐generation antipsychotics; SGAs, second‐generation antipsychotics; TD, tardive dyskinesia.

### Efficacy

**Fig. 3:**
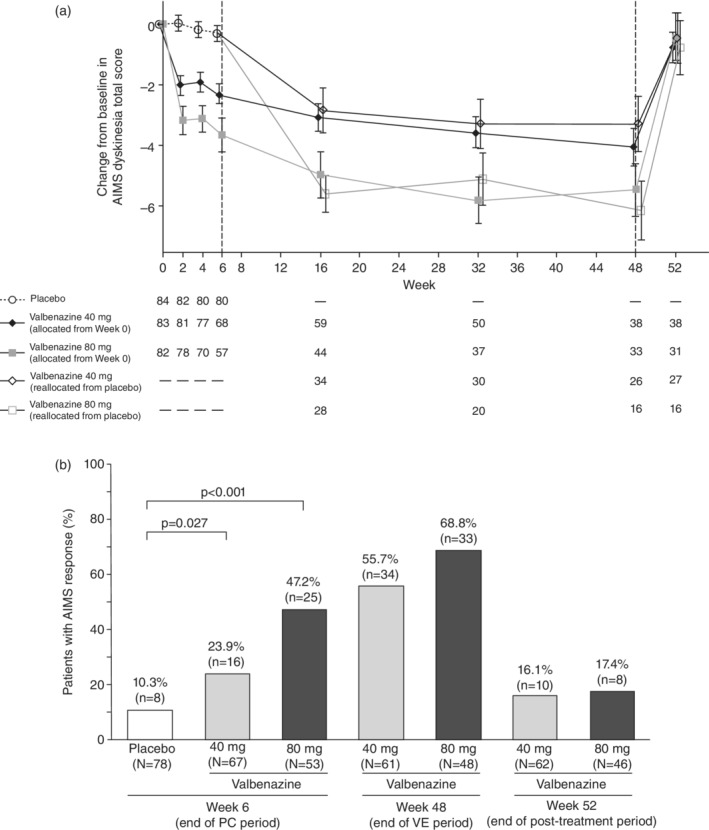
Change from baseline in AIMS total score by central assessment (A)^a^ and percentage of patients with AIMS response (B) (intention‐to‐treat analysis set). ^a^Data are means ± standard errors. AIMS, Abnormal Involuntary Movement Scale; PC, placebo‐controlled; VE, valbenazine extension.

The change from baseline in AIMS total score by central assessment (primary outcome) is shown in Fig. 3a. The least‐squares (LS) mean (95% confidence interval [CI]) of the change from baseline at Week 6 was −2.3 (−3.0, −1.7) in the valbenazine 40‐mg group, −3.7 (−4.4, −3.0) in the 80‐mg group, and − 0.1 (−0.8, 0.5) in the placebo group. Both 40‐ and 80‐mg groups showed statistically significant improvements (i.e., lower scores) *vs*. placebo: LS mean (95% CI) of the difference between valbenazine 40 mg and placebo was −2.2 (−3.0, −1.3) and the LS mean difference was −3.6 (−4.5, −2.6) for valbenazine 80 mg *vs*. placebo (both *P* < 0.001, MMRM). The mean change from baseline in the AIMS total score by central assessment decreased until the end of the VE period for patients who received 40‐ or 80‐mg valbenazine throughout the study, and returned to baseline by the end of the 4‐week post‐treatment observation period (Fig. 3a). Patients who were switched from placebo to valbenazine at Week 6 showed a similar pattern in AIMS total scores, with significant decreases throughout the VE period followed by a return to baseline post‐treatment. Similar AIMS total score results were observed by site assessment; patients receiving valbenazine had significantly better scores *vs*. placebo at Week 6, patients switched to valbenazine at Week 6 showed significant reductions in AIMS scores after switching, and both groups exhibited a sustained improvement in scores until Week 48.

The percentages of patients achieving an AIMS response, defined as ≥50% improvement from baseline in AIMS total score, are shown in Fig. 3b. The percentages of patients who met this threshold at Week 6 were 23.9%, 47.2%, and 10.3% in the 40‐mg, 80‐mg, and placebo groups, respectively. Both treatment groups had significantly higher percentages *vs*. placebo (*P* = 0.027 and *P* < 0.001 for valbenazine 40‐ and 80‐mg, respectively). The percentages of patients with an AIMS response continued to increase throughout the VE period and dropped sharply by the end of the post‐treatment observation period.

**Fig. 4:**
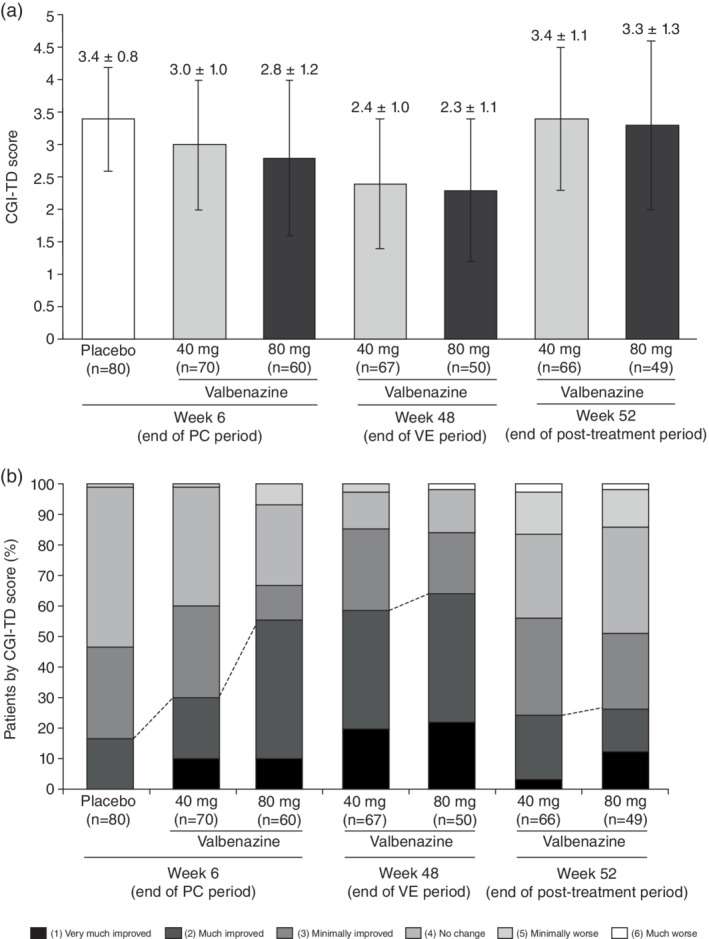
Change in CGI‐TD score throughout the study (A)^a^ and breakdown of patients by CGI‐TD categories (B)^b^ (intention‐to‐treat analysis set). ^a^Data are means ± standard deviations. ^b^No patients were assessed as “very much worse” (category 7). CGI‐TD, clinical global impression of improvement of tardive dyskinesia; PC, placebo‐controlled; VE, valbenazine extension.

The CGI‐TD results are shown in Fig. 4. The LS mean (95% CI) CGI‐TD scores at Week 6, determined by analysis of variance, were 3.0 (2.8, 3.3), 2.8 (2.5, 3.0), and 3.4 (3.2, 3.6) in the 40‐mg, 80‐mg, and placebo groups, respectively, showing statistically significant improvement for each valbenazine group *vs*. placebo: the LS mean (95% CI) of the difference between valbenazine 40 mg and placebo was −0.4 (−0.7, −0.1) (*P* = 0.021), and the LS mean difference was −0.6 (−1.0, −0.3) for valbenazine 80 mg *vs*. placebo (*P* < 0.001). CGI‐TD scores had decreased further by the end of the VE period, and returned to baseline levels at the end of the post‐treatment observation period (Fig. 4a). A breakdown of patients by CGI‐TD categories at Weeks 6, 48, and 52 is illustrated in Fig. 4b.

### Safety

During the PC period, 54/85 (63.5%), 67/84 (79.8%), and 37/84 (44.0%) patients experienced TEAEs in the valbenazine 40‐mg, valbenazine 80‐mg, and placebo groups, respectively (Table 2). During the long‐term treatment period, TEAEs were reported in 113/126 (89.7%) and 116/123 (94.3%) patients in the valbenazine 40‐ and 80‐mg groups, respectively. The incidence of TEAEs and related TEAEs leading to discontinuation or dose reduction was higher in the 80‐mg group than the 40‐mg group; however, the incidence of serious TEAEs was not significantly different between valbenazine treatment groups during the long‐term treatment period.

**Table 2:** Incidence of TEAEs and related TEAEs during the 6‐week placebo‐controlled period; long‐term treatment period; and 4‐week post‐treatment observational period (safety analysis set)

PC period				
	Placebo (*n* = 84)	Valbenazine		
		40 mg (*n* = 85)	80 mg (*n* = 84)	Total (*N* = 169)
Patients with ≥1 TEAE	37 (44.0)	54 (63.5)	67 (79.8)	121 (71.6)
≥1 serious TEAE	1 (1.2)	4 (4.7)	1 (1.2)	5 (3.0)
≥1 TEAE leading to treatment discontinuation	3 (3.6)	6 (7.1)	14 (16.7)	20 (11.8)
≥1 TEAE leading to dose reduction	0 (0.0)	1 (1.2)	10 (11.9)	11 (6.5)
TEAE resulting in death	0 (0.0)	0 (0.0)	0 (0.0)	0 (0.0)
Patients with ≥1 related TEAE	11 (13.1)	28 (32.9)	46 (54.8)	74 (43.8)
≥1 serious related TEAE	0 (0.0)	0 (0.0)	0 (0.0)	0 (0.0)
≥1 related TEAE leading to treatment discontinuation	2 (2.4)	4 (4.7)	10 (11.9)	14 (8.3)
≥1 related TEAE leading to dose reduction	0 (0.0)	1 (1.2)	10 (11.9)	11 (6.5)

Long‐term treatment period				
		Valbenazine		
		40 mg (*n* = 126)	80 mg (*n* = 123)	Total (*N* = 249)
Patients with ≥1 TEAE	—	113 (89.7)	116 (94.3)	229 (92.0)
≥1 serious TEAE	—	18 (14.3)	16 (13.0)	34 (13.7)
≥1 TEAE leading to treatment discontinuation	—	23 (18.3)	36 (29.3)	59 (23.7)
≥1 TEAE leading to dose reduction	—	16 (12.7)	39 (31.7)	55 (22.1)
TEAE resulting in death	—	3 (2.4)	4 (3.3)	7 (2.8)
Patients with ≥1 related TEAE	—	64 (50.8)	92 (74.8)	156 (62.7)
≥1 serious related TEAE	—	2 (1.6)	7 (5.7)	9 (3.6)
≥1 related TEAE leading to treatment discontinuation	—	12 (9.5)	24 (19.5)	36 (14.5)
≥1 related TEAE leading to dose reduction	—	15 (11.9)	38 (30.9)	53 (21.3)

Post‐treatment observation period				
		Valbenazine		
		40 mg (*n* = 67)	80 mg (*n* = 50)	Total (*N* = 117)
Patients with ≥1 TEAE	—	16 (23.9)	13 (26.0)	29 (24.8)
≥1 serious TEAE	—	1 (1.5)	2 (4.0)	3 (2.6)
≥1 TEAE leading to treatment discontinuation	—	0 (0.0)	0 (0.0)	0 (0.0)
≥1 TEAE leading to dose reduction	—	0 (0.0)	0 (0.0)	0 (0.0)
TEAE resulting in death^a^	—	0 (0.0)	0 (0.0)	0 (0.0)
Patients with ≥1 related TEAE	—	0 (0.0)	4 (8.0)	4 (3.4)
≥1 serious related TEAE	—	0 (0.0)	1 (2.0)	1 (0.9)
≥1 related TEAE leading to treatment discontinuation	—	0 (0.0)	0 (0.0)	0 (0.0)
≥1 related TEAE leading to dose reduction	—	0 (0.0)	0 (0.0)	0 (0.0)

^a^ Does not include one patient who died during follow‐up after discontinuation of the study drug.

Data are *n* (%). Related, possibly or definitely related to the study drug.

TEAE, treatment‐emergent adverse event; PC, placebo‐controlled.

TEAEs occurring in ≥2% of patients in the valbenazine total group (i.e., the 40‐ and 80‐mg groups combined), who were treated with valbenazine during the PC period, and TEAEs occurring in ≥5% of patients in either treatment group during the long‐term treatment period are listed in Table S1. Somnolence, hypersalivation, malaise, worsening of schizophrenia, and tremor were notably more common in patients who received valbenazine than in those who received placebo during the PC period. During the long‐term treatment period, TEAEs occurring at a rate of ≥10% in either valbenazine group included nasopharyngitis, somnolence, worsening of schizophrenia, hypersalivation, insomnia, and tremor. No serious related TEAEs or deaths occurred during the PC period. During the long‐term treatment period, serious TEAEs that occurred in two or more patients included worsening of schizophrenia, depression, and death; the only serious related TEAEs that occurred in two or more patients was worsening of schizophrenia. TEAEs related to schizophrenia, depression, and other psychiatric disorders were reported in this study. However, scores obtained using the psychiatric symptom rating scales (JCDSS, MADRS‐J, YMRS, and PANSS) showed no noteworthy changes at either the 6‐ or 48‐week timepoint (Tables S2 and S3), thus showing that the patients' psychiatric condition was stable throughout the study.

Seven patients died during the study (three in the valbenazine 40‐mg group and four in the 80‐mg group); the mean age of the patients who died was 69.4 years, and five were male. One additional patient (originally in the 80‐mg group) died after discontinuation of the study drug. TEAEs leading to death were death (i.e., cause unknown; *n* = 2), respiratory failure, myocardial ischemia, pneumonia, aspiration pneumonia, marasmus, and acute hepatic failure (*n* = 1 each). All deaths were ruled by the investigator to have no plausible relationship to the study drug, except for one death in the 40‐mg group for which a cause could not be determined. The investigators judged that the 20 other medications used by this patient may have contributed to the death. Additional details on TEAEs leading to death are described in Appendix S1.

No notable changes were observed in vital signs, 12‐lead electrocardiograms, or laboratory test values, except for serum prolactin level. The mean prolactin level in each valbenazine group increased after Week 2 of treatment; this elevation was sustained throughout the treatment period and then decreased to baseline level 4 weeks after treatment completion.

## Discussion

This multicenter, phase II/III, randomized, double‐blind, placebo‐controlled study assessed the efficacy and safety of valbenazine for treatment of TD in psychiatric patients in Japan. Significant improvements in the primary and secondary efficacy endpoints (AIMS total score and CGI‐TD) demonstrated that valbenazine effectively improved symptoms of TD. The incidence of TEAEs was higher in the valbenazine groups than for placebo, and the incidence of TEAEs such as somnolence, hypersalivation, and tremor was particularly high in the valbenazine 80‐mg group, although most of these events were mild or moderate in severity. A total of seven deaths were reported, and a causal relationship to the study drug was ruled out in all but one, a patient with depressive disorder and multiple comorbidities, who was found collapsed in front of her home.

Before 2017, there was no treatment for TD supported by sufficient evidence. However, publication in 2017 of the findings of two studies^12^, ^13^ demonstrating the clinical usefulness of VMAT2 inhibitors for this indication resulted in changes to the treatment algorithm for TD in North America.^27^ Our study is the first to demonstrate the efficacy and safety of a VMAT2 inhibitor in a trial conducted outside of North America and Europe, where previous trials were mainly conducted, and supports the generalizability of the findings of the previous studies.^12^, ^13^

The prevalence of TD has been reported to vary by region: from 17.3% in Asia, to 23.3% in Europe, to 31.3% in the US among psychiatric patients receiving treatment with antipsychotics.^28^ Regarding ethnicity, African Americans have been reported as being at higher risk of developing TD than other races.^29^ Because of such differences across regions and ethnic groups, the generalizability of any treatment for TD needs to be established. However, to date, no reports have provided strong evidence for the efficacy of any TD treatment in Asian populations. Against this background, our results, showing the efficacy and safety of VMAT2 inhibitors in an Asian population, are of great significance.

The results of the present study were likely consistent with those of previous studies^12^, ^17^ owing to the following aspects of study design.^30^ AIMS assessment was performed at a central assessment facility, using video recordings obtained at the study sites and handled in a blinded manner (in terms of treatment and evaluation time point). Central assessment may be a key factor in ensuring objective and empirical assessment of results of clinical trials for treatments for TD.^30^ In our study, the efficacy of valbenazine as demonstrated by the centrally performed AIMS assessments was supported by the results of CGI‐TD assessments performed at the study sites.

The phase III KINECT‐3 study conducted in the US and Canada recruited a similar number of patients, had a similar study design to the present study, and had similar results with respect to efficacy (AIMS and CGI‐TD scores).^12^, ^17^ The LS mean changes from baseline in AIMS total score at Week 6 in the present study were − 2.3 and − 3.7 in the 40‐ and 80‐mg groups, respectively, compared with −1.9 and − 3.2 at Week 6 in KINECT‐3.^12^ The mean ± standard error changes in AIMS total score from baseline to Week 48 in the present study were − 3.7 ± 4.2 and − 5.7 ± 4.6 in the 40‐ and 80‐mg groups, respectively, compared with mean changes of −3.0 and − 4.8 at Week 48 in KINECT‐3.^17^ CGI‐TD scores also improved across studies, with the LS mean scores consistently lower (i.e., representing improvement) among patients who received valbenazine: Week 6 scores in the present study were 3.4, 3.0, and 2.8 for placebo, valbenazine 40 mg, and 80 mg, respectively. In the present study, the respective mean ± SD CGI‐TD scores in the 40‐ and 80‐mg groups were 2.4 ± 1.0 and 2.3 ± 1.1 at Week 48, and 3.4 ± 1.1 and 3.3 ± 1.3 at Week 52. In KINECT‐3, statistically significant improvement *vs*. placebo was observed in the per‐protocol set at Week 6 (3.2, 2.8, 2.8 for placebo, valbenazine 40 mg, and valbenazine 80 mg, respectively); further improvement was achieved during the long‐term extension phase, with scores dropping to 2.4 and 2.1 at Week 48 for patients who received 40‐ and 80‐mg valbenazine, respectively, then rebounding to 3.1 and 3.5 by the end of the 4‐week washout period (Week 52).

However, when comparing safety results between KINECT‐3 and the present study, the incidences of TEAEs for valbenazine were lower in KINECT‐3 than in our study: 40.3% *vs*. 63.5% for valbenazine 40 mg, 50.6% *vs*. 79.8% for valbenazine 80 mg, and 43.4% *vs*. 44.0% for placebo in the PC period. In KINECT‐3, there was no significant difference in the incidence of TEAEs between valbenazine dose groups; however, the incidence of TEAEs was higher in the 80‐mg group than the 40‐mg group in our study. Although the reason for the relatively high incidence of TEAEs in our study is not clear, it is important to note that there was no significant difference in the incidence of severe TEAEs between the KINECT‐3 study and our study, and we believe our results demonstrate a favorable risk‐benefit balance with respect to treatment of Japanese patients. Common TEAEs observed in our study such as somnolence, tremor, and hypersalivation are thought to be caused by the pharmacological effects of VMAT2 inhibitors (i.e., a decrease in monoamine transporter activity), and the incidence of such events might be expected to increase with increased valbenazine exposure. However, we did not find any differences in the incidence of TEAEs according to patient age, body mass index, or other factors, and thus the patient background factors that may explain the differences between results in Japanese and non‐Japanese studies remain unknown.

Valbenazine is approved in the US for the treatment of TD and is recommended for this indication by the practice guidelines of the American Psychiatric Association.^27^ In Japan, however, valbenazine has only just been approved for the treatment of TD as of March 2022, and is not yet recommended in the guidelines. Current Japanese practice guidelines recommend dose reduction or discontinuation of the antipsychotic medication causing extrapyramidal symptoms in psychiatric patients.^31^ However, the efficacy of dose reduction or discontinuation of antipsychotic medication has not been adequately established and may not be possible in some patients, as worsening of psychotic symptoms may occur.

Consistent with previous studies, the present study showed that patients' psychiatric condition remains stable when a VMAT2 inhibitor is added to their antipsychotic medication. This finding adds support for VMAT2 inhibitors as a promising option for psychiatric patients with TD; not only do VMAT2 inhibitors enable control of TD symptoms without necessitating changes to pharmacotherapy for the underlying psychiatric condition, but they do so without changes in psychiatric stability.

Compared with other VMAT2 inhibitors (tetrabenazine and deutetrabenazine), valbenazine has a longer half‐life, allowing once‐daily administration; this is more convenient for patients, particularly those taking multiple medications.^32^, ^33^ Furthermore, as valbenazine has only a single metabolite that is highly selective for VMAT2, it is expected to have fewer off‐target effects than other VMAT2 inhibitors, whose multiple metabolites have some affinity for serotonin and dopamine D2 receptors.^34^

Our study was limited in that the placebo‐controlled period was relatively short (6 weeks), however, the efficacy during the 42‐week extension period was similar to the placebo‐controlled period. We included only psychiatric patients with stable symptoms and stable maintenance therapy for the underlying disease; therefore, the usefulness of valbenazine in patients with unstable disease is not known.

In Japan, the lack of effective treatments for TD was a significant unmet clinical need of patients who experience this often‐persistent side effect of antipsychotic medications. Our study demonstrated that valbenazine improved TD symptoms without affecting the underlying psychiatric condition and there were no major concerns about safety/tolerability. Valbenazine may be a safe and effective treatment option for Japanese psychiatric patients who experience TD.

## Disclosure statement

Dr. Horiguchi has received manuscript fees from Mitsubishi Tanabe Pharma Corp.; and is a consultant for Mitsubishi Tanabe Pharma Corp.

Dr. K Watanabe has received manuscript fees or speaker's honoraria from Eisai Co. Ltd., Janssen Pharmaceutical K.K., Kyowa Pharmaceutical Industry Co. Ltd., Eli Lilly Japan K.K., Lundbeck Japan, Meiji Seika Pharma Co. Ltd., Mitsubishi Tanabe Pharma Corp., MSD K.K., Otsuka Pharmaceutical Co., Pfizer Japan Inc., Shionogi Inc., Sumitomo Dainippon Pharma Co., Ltd., and Takeda Pharmaceutical Co., Ltd.; research and grant support from Daiichi Sankyo Co. Ltd., Eisai Co. Ltd., Meiji Seika Pharma Co., Ltd., Mitsubishi Tanabe Pharma Corp., MSD K.K., Otsuka Pharmaceutical Co., Ltd., Pfizer Japan Inc., Sumitomo Dainippon Pharma Co., Ltd. and Takeda Pharmaceutical Co., Ltd.; and consulting fees from Boehringer Ingelheim, Daiichi Sankyo Co. Ltd., Eisai Co. Ltd., Janssen Pharmaceutical K.K., Kyowa Pharmaceutical Industry Co. Ltd., Eli Lilly Japan K.K., Lundbeck Japan, Luye Pharma, Mitsubishi Tanabe Pharma Corp., Otsuka Pharmaceutical Co., Ltd., Pfizer Japan Inc., Sumitomo Dainippon Pharma Co., Ltd., and Taisho Toyama Pharmaceutical Co. Ltd., and Takeda Pharmaceutical Co., Ltd.

Drs Kondo, Iwatake, Sakamoto, Susuta, Masui, and Y Watanabe are employees of Mitsubishi Tanabe Pharma Corp., which funded this study.

## Author contributions

JH, KW, and KK supervised the design and protocol of the study and contributed to the interpretation and discussion of the results as medical advisors. AI and HS contributed to the study design and collected the data. YS contributed to the study design, data processing, and statistical analysis. HM and YW contributed to the writing of the manuscript. All authors contributed to the discussion of the results, reviewed the manuscript, and approved the final version of the manuscript.

## Data Sharing Statement

The deidentified datasets generated and/or analyzed during this study including protocols, annotated case report form, dataset specifications, and clinical study report may be available from Mitsubishi Tanabe Pharma Corporation (MTPC) *via* the corresponding author if MTPC agrees on the proposer's request after internal review.

Once MTPC gives approval for the proposal, MTPC will execute a Data Sharing Agreement with the proposer. As a next step, MTPC will disclose clinical data for this study. The request must not extend beyond the limitation of the participants' informed consent. External research is limited to the conduct of approved research according to a Data Sharing Agreement with MTPC.

Access to data may be declined by MTPC when there is a potential conflict of interest, or an actual or potential competitive risk, and/or other conditions with its existing partner. In addition, access to data will not be shared until 3 years after the article is published at the earliest timepoint.

## Field

Neuropsychopharmacology (primary); Clinical neurophysiology and neuropsychology (secondary).

## Previous Presentation

Some of the results described in this paper were presented at the 31st Annual Meeting of The Japanese Society of Clinical Neuropsychopharmacology, Tokyo, Japan (October 7–8, 2021). Some of the results described in this paper will be presented at the 118th Annual Meeting of the Japanese Society of Psychiatry and Neurology, Fukuoka, Japan (June 16–18, 2022).

## Supporting information


**Appendix S1.** Supplementary methods
**Table S1.** Treatment‐emergent adverse events occured in ≥2% of patients in the valbenazine total group during the placebo‐controlled period, and in ≥5% of patients in either treatment group during the long‐term period (safety analysis set).
**Table S2.** Changes in underlying psychiatric disease at Week 6 (end of placebo‐controlled period) (safety analysis set).
**Table S3.** Changes in underlying psychiatric disease at Week 48 (end of long‐term period) (safety analysis set).
