# Supplementary material for: Efficacy and safety of valbenazine in Japanese patients with tardive dyskinesia: A multicenter, randomized, double‐blind, placebo‐controlled study (J‐KINECT)
Source: Psychiatry Clin Neurosci. 2022 Sep 17;76(11):560–9. doi: 10.1111/pcn.13455 (PMC9826124; doi:10.1111/pcn.13455)
Supplement: Supplementary file 1 — Appendix S1. Supplementary methods Table S1. Treatment‐emergent adverse events occured in ≥2% of patients in the valbenazine total group during the placebo‐controlled period, and in ≥5% of patients in either treatment group during the long‐term period (safety analysis set). Table S2. Changes in underlying psychiatric disease at Week 6 (end of placebo‐controlled period) (safety analysis set). Table S3. Changes in underlying psychiatric disease at Week 48 (end of long‐term period) (safety analysis set). [file PCN-76-560-s001.docx]

**Supporting information**

**Supplementary methods**

**1. Full list of exclusion criteria**

Patients who met any of the following criteria were excluded from the study:

(1) Active, clinically significant, unstable cerebrovascular disease, hepatic disease, renal disease, endocrine disease, cardiovascular disease, gastrointestinal disease, respiratory disease, or metabolic disease during the pre-treatment observation period.

(2) Dementia diagnosis according to the diagnostic criteria of the DSM-5 during the pre-treatment observation period.

(3) Presence of prominent abnormal involuntary movements, such as coexisting dystonia, akathisia, or parkinsonism, and TD that cannot be properly evaluated by AIMS central assessment based on recorded video at screening.

(4) Simpson–Angus Extrapyramidal Side Effects Scale (SAS) score of ≥3 at baseline in ≥2 items (except items 8 and 10).

(5) Presence of substance-related disorder (except tobacco- or caffeine-related disorder) according to the diagnostic criteria of the DSM-5 within 3 months before initial randomization.

(6) High risk of suicidal or self-injurious behavior as judged by the investigator (or subinvestigator), or suicide attempt or suicidal ideation corresponding to item 4 or 5 on the Columbia Suicide Severity Rating Scale (C-SSRS) assessment at baseline (within 3 months before evaluation).

(7) History of neuroleptic malignant syndrome within 3 years before initial randomization.

(8) History of long QT syndrome or tachyarrhythmia within 3 years before initial randomization.

(9) Fridericia’s correction of QT interval (QTcF) ≥450 ms (men) or ≥470 ms (women) on a standard 12-lead electrocardiogram at screening.

(10) Abnormalities in the following laboratory tests at screening:

(a) Serum creatinine >1.5 × upper limit of normal (ULN)

(b) Alanine aminotransferase or aspartate aminotransferase ≥2.5 × ULN

(c) γ-Glutamyl transpeptidase ≥ 3 × ULN*

(d) Total bilirubin >1.5 mg/dL

(e) Hemoglobin <10 g/dL

(f) White blood cell count <3,000/mm^3^

(g) Platelet count <100,000/mm^3^

**Protocol amendment:* Exceptions may be made in accordance with the approval of the sponsor’s medical expert.

(11) History of malignant tumor within 3 years before initial randomization. However, complete excision of basal cell carcinoma or squamous cell carcinoma of the skin is excluded.

(12) Positive for hepatitis B antigen, hepatitis C antibody,* or HIV antibody at screening **Protocol amendment:* HCV antibody-positive patients might be enrolled only if RNA testing is negative.

(13) Administration of another investigational product within 6 months before initial randomization.

(14) Previous treatment with deep brain stimulation within 3 years before initial randomization.

(15) History of surgery known to affect digestive tract absorption of drugs.

(16) History of drug allergy*** or previous tetrabenazine administration

**Protocol amendment:* Patients with a history of drug allergy may be enrolled, subject to the approval of the sponsor’s medical expert.

(17) Unwillingness to use appropriate contraception from the time of informed consent until 28 days after the completion (discontinuation) of study treatment.

(18) Pregnancy, breastfeeding, or possible pregnancy.

(19) Judged as ineligible for this study by the investigator or subinvestigator.

For patients with schizophrenia or schizoaffective disorder:

(20) Calgary Depression Scale for Schizophrenics, Japanese Version (JCDSS) total score of ≥10 at baseline.

(21) Positive and Negative Syndrome Scale (PANSS) total score of ≥70 at baseline.

For patients with bipolar disorder or depressive disorder:

(22) Hospitalized for treatment of bipolar disorder or major depressive disorder within 6 months before initial randomization.

(23) Occurrence of a mood episode (manic symptoms or depressive symptoms) within 3 months before initial randomization.

(24) History of rapid cycling (>4 episodes per year) or ultra-rapid cycling (>4 episodes per month).

(25) Montgomery–Asberg Depression Rating Scale, Japanese Version (MADRS-J) total score of >13 at baseline.

(26) Young Mania Rating Scale (YMRS) total score of >10 at baseline.

**2. Restrictions on concomitant medications/therapies.**

Regarding restrictions on concomitant medications/therapies, the dosage regimen for maintenance therapy of the underlying psychiatric disorder was not to be changed within 30 days prior to the initial observation period (14 days for benzodiazepines), until the end of the post-treatment observation period, unless unavoidable for treatment of adverse events. Initiation or discontinuation of drugs for treatment of the underlying psychiatric disorder was not permitted during the study. Benzodiazepines were permitted as maintenance therapy or as sleeping aids. Use of hypnotics was permitted, provided abnormal involuntary movement scale (AIMS) assessments were performed at least 8 hours after administration.

Botulinum toxin injection was prohibited from 90 days prior to study initiation. For 30 days prior to study initiation, drugs and food with potent cytochrome P450 (CYP) 3A4 inhibitory or inducing action, potent CYP2D6 inhibitors, antiemetics, dopamine agonists/precursors, monoamine oxidase inhibitors, central nervous system stimulants, VMAT-2 inhibitors (other than the investigation product), deep brain stimulation, *Ginkgo biloba*, vitamin E supplements, and yokukansan were prohibited.

**3. Statistical analysis for secondary endpoints.**

The percentage of AIMS responders at Week 6 was compared between each of the treatment groups and placebo using the Cochran–Mantel–Haenszel test with underlying disease as a stratification factor. Descriptive statistics for CGI-TD score at Week 6 were calculated for each treatment group. Analysis of variance was performed with the group and underlying disease as fixed effects.

**Details of patients who died during the study**

Of the seven patients who died during the study, six cases were ruled by the investigator to have no plausible relationship to the study drug. One death in the 40-mg group, for which a cause could not be determined, was ruled as having a possible relationship to the study drug, although the investigators noted that the 20 other medications used by this patient may have contributed to the death.

Causality for the remaining six patients who died during the study was assessed as follows:

1.Unknown: Classed as unknown cause of death because, although supraventricular extrasystoles had occurred, the patient had no subjective symptoms and had recovered from coughing, fatigue, and somnolence prior to death. The death was determined to be an incidental event and not causally related to the study drug.

2. Respiratory failure: Judged as caused by pre-existing asthma comorbidity and no plausible relationship to the study drug.

3. Myocardial ischemia: Judged as owing to pre-existing cardiac condition and no plausible relationship to the study drug.

4.Pneumonia: Judged to be caused by a decrease in fitness owing to poor eating as a result of bipolar disorder and no plausible relationship to the study drug.

5. Aspiration pneumonia: Judged as unrelated to the study drug because aspiration pneumonia had previously also occurred prior to study participation due to pre-existing dysphagia.

6. Marasmus: Judged as being caused by poor eating due to oral discomfort as a result of tardive dyskinesia and therefore unrelated to the study drug.

**Details of the patient who died after discontinuation of the study drug**

In addition to the seven patients who died during the study, one patient died after discontinuation of the study drug. In this patient, causality was assessed as follows.

Acute hepatic failure: Judged as unrelated to the study drug because there was no evidence of hepatic dysfunction during the study treatment period and the event occurred 2 weeks after completion of treatment.

**Table S1.** Treatment-emergent adverse events occurring in ≥2% of patients in the valbenazine total group during the placebo-controlled period, and in ≥5% of patients in either treatment group during the long-term period (safety analysis set).

| **PC period** | | | | |
| --- | --- | --- | --- | --- |
|  |  | **Valbenazine** | | |
|  | **Placebo**  **(n=84)** | **40 mg**  **(n=85)** | **80 mg**  **(n=84)** | **Total**  **(N=169)** |
| All TEAEs | 37 (44.0) | 54 (63.5) | 67 (79.8) | 121 (71.6) |
| Infections and infestations | 7 (8.3) | 10 (11.8) | 9 (10.7) | 19 (11.2) |
| Nasopharyngitis | 6 (7.1) | 6 (7.1) | 4 (4.8) | 10 (5.9) |
| Psychiatric disorders | 5 (6.0) | 17 (20.0) | 16 (19.0) | 33 (19.5) |
| Insomnia | 1 (1.2) | 2 (2.4) | 5 (6.0) | 7 (4.1) |
| Schizophrenia | 1 (1.2) | 7 (8.2) | 0 (0.0) | 7 (4.1) |
| Anxiety | 0 (0.0) | 1 (1.2) | 4 (4.8) | 5 (3.0) |
| Depression | 1 (1.2) | 1 (1.2) | 3 (3.6) | 4 (2.4) |
| Nervous system disorders | 6 (7.1) | 20 (23.5) | 38 (45.2) | 58 (34.3) |
| Somnolence | 2 (2.4) | 10 (11.8) | 21 (25.0) | 31 (18.3) |
| Akathisia | 1 (1.2) | 4 (4.7) | 5 (6.0) | 9 (5.3) |
| Headache | 2 (2.4) | 1 (1.2) | 4 (4.8) | 5 (3.0) |
| Tremor | 0 (0.0) | 0 (0.0) | 5 (6.0) | 5 (3.0) |
| Gastrointestinal disorders | 7 (8.3) | 8 (9.4) | 20 (23.8) | 28 (16.6) |
| Salivary hypersecretion | 1 (1.2) | 3 (3.5) | 9 (10.7) | 12 (7.1) |
| Skin and subcutaneous tissue disorders | 5 (6.0) | 6 (7.1) | 6 (7.1) | 12 (7.1) |
| Urticaria | 0 (0.0) | 2 (2.4) | 2 (2.4) | 4 (2.4) |
| Musculoskeletal and connective tissue disorders | 5 (6.0) | 4 (4.7) | 5 (6.0) | 9 (5.3) |
| Back pain | 2 (2.4) | 3 (3.5) | 2 (2.4) | 5 (3.0) |
| General disorders and administration site conditions | 2 (2.4) | 10 (11.8) | 11 (13.1) | 21 (12.4) |
| Malaise | 0 (0.0) | 5 (5.9) | 4 (4.8) | 9 (5.3) |
| **Long-term treatment period** | | | | |
|  |  | **Valbenazine** | | |
|  |  | **40 mg**  **(n=126)** | **80 mg**  **(n=123)** | **Total**  **(N=249)** |
| All TEAEs | — | 113 (89.7) | 116 (94.3) | 229 (92.0) |
| Infections and infestations | — | 46 (36.5) | 40 (32.5) | 86 (34.5) |
| Nasopharyngitis | — | 30 (23.8) | 23 (18.7) | 53 (21.3) |
|  |  |  |  |  |
| Psychiatric disorders | — | 52 (41.3) | 57 (46.3) | 109(43.8) |
| Schizophrenia | — | 21 (16.7) | 13 (10.6) | 34 (13.7) |
| Insomnia | — | 10 (7.9) | 16 (13.0) | 26 (10.4) |
| Depression | — | 6 (4.8) | 10 (8.1) | 16 (6.4) |
| Anxiety | — | 5 (4.0) | 9 (7.3) | 14 (5.6) |
| Nervous system disorders | — | 51 (40.5) | 69 (56.1) | 120 (48.2) |
| Somnolence | — | 19 (15.1) | 28 (22.8) | 47 (18.9) |
| Tremor | — | 5 (4.0) | 16 (13.0) | 21 (8.4) |
| Akathisia | — | 8 (6.3) | 12 (9.8) | 20 (8.0) |
| Headache | — | 4 (3.2) | 8 (6.5) | 12 (4.8) |
| Dizziness | — | 4 (3.2) | 7 (5.7) | 11 (4.4) |
| Gastrointestinal disorders | — | 39 (31.0) | 51 (41.5) | 90 (36.1) |
| Salivary hypersecretion | — | 9 (7.1) | 21 (17.1) | 30 (12.0) |
| Constipation | — | 9 (7.1) | 9 (7.3) | 18 (7.2) |
| Musculoskeletal and connective tissue disorders | — | 27 (21.4) | 23 (18.7) | 50 (20.1) |
| Back pain | — | 7 (5.6) | 6 (4.9) | 13 (5.2) |
| General disorders and administration site conditions | — | 27 (21.4) | 25 (20.3) | 52 (20.9) |
| Malaise | — | 10 (7.9) | 10 (8.1) | 20 (8.0) |
| Thirst | — | 7 (5.6) | 2 (1.6) | 9 (3.6) |
| Injury, poisoning and procedural complications | — | 21 (16.7) | 22 (17.9) | 43 (17.3) |
| Contusion | — | 5 (4.0) | 7 (5.7) | 12 (4.8) |
| Fall | — | 2 (1.6) | 7 (5.7) | 9 (3.6) |

Data are n (%).

TEAEs are coded by System Organ Class and Preferred Term, using the Medical Dictionary for Regulatory Activities, Japanese version 23.0.

TEAE, treatment-emergent adverse event; PC, placebo-controlled

**Table S2.** Changes in underlying psychiatric disease at Week 6 (end of placebo-controlled period) (safety analysis set)

|  | |  | **Valbenazine** | | | |
| --- | --- | --- | --- | --- | --- | --- |
|  |  | **Placebo**  **(n=84)** | **40 mg (n=85)** | **80 mg (n=84)** | **Total (N=169)** | |
| JCDSS total score | Baseline | 2.4 ± 2.6  (n=53) | 2.8 ± 2.3  (n=55) | 2.7 ± 2.6  (n=54) | 2.8 ± 2.5  (n=109) | |
|  | Week 6 | 2.3 ± 3.0  (n=52) | 2.8 ± 3.3  (n=46) | 2.8 ± 2.6  (n=40) | 2.8 ± 3.0  (n=86) | |
|  | Change | −0.1 ± 1.7 | 0.0 ± 3.3 | 0.1 ± 2.2 | 0.1 ± 2.9 | |
| MADRS-J total score | Baseline | 5.2 ± 4.0  (n=31) | 5.9 ± 4.2  (n=30) | 5.9 ± 3.9  (n=30) | 5.9 ± 4.0  (n=60) | |
|  | Week 6 | 5.1 ± 5.2  (n=28) | 6.3 ± 6.1  (n=24) | 7.0 ± 6.1  (n=20) | 6.6 ± 6.0  (n=44) | |
|  | Change | 0.3 ± 4.6 | 0.7 ± 4.5 | 0.8 ± 5.3 | 0.7 ± 4.8 | |
| YMRS total score | Baseline | 1.1 ± 1.9  (n=31) | 1.3 ± 1.9  (n=30) | 1.4 ± 1.7  (n=30) | 1.4 ± 1.8  (n=60) | |
|  | Week 6 | 0.8 ± 1.8  (n=28) | 0.3 ± 0.6  (n=24) | 1.8 ± 3.1  (n=20) | 1.0 ± 2.3  (n=44) | |
|  | Change | −0.3 ± 1.6 | −0.6 ± 1.2 | 0.0 ± 2.2 | −0.3 ± 1.7 | |
| PANSS | | | | | |  |
| Positive scale | Baseline | 11.6 ± 3.4  (n=53) | 11.7 ± 4.0  (n=55) | 11.2 ± 3.0  (n=54) | 11.5 ± 3.5  (n=109) | |
|  | Week 6 | 11.2 ± 3.4  (n=52) | 11.8 ± 3.4  (n=46) | 11.5 ± 3.6  (n=39) | 11.7 ± 3.5  (n=85) | |
|  | Change | −0.5 ± 1.8 | 0.2 ± 2.5 | 0.0 ± 1.3 | 0.1 ± 2.1 | |
| Negative scale | Baseline | 17.0 ± 4.2  (n=53) | 16.3 ± 4.8  (n=55) | 15.9 ± 5.1  (n=54) | 16.1 ± 4.9  (n=109) | |
|  | Week 6 | 16.5 ± 4.7  (n=52) | 16.2 ± 4.2  (n=46) | 16.1 ± 5.1  (n=39) | 16.2 ± 4.6  (n=85) | |
|  | Change | −0.5 ± 2.5 | 0.4 ± 2.2 | −0.1 ± 2.8 | 0.2 ± 2.5 | |
| General psychopathology scale | Baseline | 28.6 ± 5.2  (n=53) | 29.2 ± 6.8  (n=55) | 27.1 ± 6.4  (n=54) | 28.2 ± 6.6  (n=109) | |
|  | Week 6 | 27.9 ± 5.5  (n=52) | 29.4 ± 6.7  (n=46) | 28.2 ± 6.5  (n=39) | 28.9 ± 6.6  (n=85) | |
|  | Change | −0.8 ± 3.4 | 0.5 ± 5.6 | 0.5 ± 3.3 | 0.5 ± 4.7 | |
| Composite scale | Baseline | −5.4 ± 5.1  (n=53) | −4.6 ± 5.2  (n=55) | −4.6 ± 5.8  (n=54) | −4.6 ± 5.4  (n=109) | |
|  | Week 6 | −5.3 ± 5.0  (n=52) | −4.4 ± 4.6  (n=46) | −4.6 ± 5.9  (n=39) | −4.5 ± 5.2  (n=85) | |
|  | Change | 0.0 ± 2.9 | −0.2 ± 2.8 | 0.1 ± 2.9 | 0.0 ± 2.8 | |

Data are mean ± standard deviation.

JCDSS, Calgary Depression Scale for Schizophrenia, Japanese version; MADRS-J, Montgomery–Asberg Depression Rating Scale, Japanese version; PANSS, positive and negative symptom scale; YMRS, Young Mania Rating Scale

**Table S3.** Changes in underlying psychiatric disease at Week 48 (end of long-term period) (safety analysis set)

|  | | **Valbenazine** | | |
| --- | --- | --- | --- | --- |
|  |  | **40 mg**  **(n=126)** | **80 mg**  **(n=123)** | **Total**  **(N=249)** |
| JCDSS total score | Baseline | 2.7 ± 2.4  (n=82) | 2.6 ± 2.6  (n=79) | 2.6 ± 2.5  (n=161) |
|  | Week 48 | 2.0 ± 3.7  (n=42) | 2.0 ± 2.7  (n=35) | 2.0 ± 3.3  (n=77) |
|  | Change | −0.2 ± 3.4 | −0.3 ± 2.3 | −0.2 ± 3.0 |
| MADRS-J total score | Baseline | 5.4 ± 4.1  (n=44) | 5.6 ± 3.9  (n=44) | 5.5 ± 3.9  (n=88) |
|  | Week 48 | 5.2 ± 6.1  (n=25) | 8.2 ± 9.9  (n=15) | 6.3 ± 7.7  (n=40) |
|  | Change | 0.4 ± 5.4 | 2.2 ± 8.2 | 1.1 ± 6.5 |
| YMRS total score | Baseline | 1.6 ± 2.1  (n=44) | 1.0 ± 1.6  (n=44) | 1.3 ± 1.9  (n=88) |
|  | Week 48 | 0.9 ± 3.2  (n=25) | 0.7 ± 1.0  (n=15) | 0.8 ± 2.6  (n=40) |
|  | Change | −0.2 ± 3.6 | −0.7 ± 1.7 | −0.4 ± 3.0 |
| PANSS | | | | |
| Positive scale | Baseline | 11.7 ± 3.9  (n=82) | 11.4 ± 3.1  (n=79) | 11.6 ± 3.5  (n=161) |
|  | Week 48 | 11.8 ± 4.0  (n=42) | 11.3 ± 4.3  (n=35) | 11.6 ± 4.1  (n=77) |
|  | Change | 0.2 ± 3.0 | −0.1 ± 3.4 | 0.1 ± 3.2 |
| Negative scale | Baseline | 16.7 ± 4.5  (n=82) | 16.1 ± 5.0  (n=79) | 16.4 ± 4.7  (n=161) |
|  | Week 48 | 17.2 ± 5.8  (n=42) | 17.7 ± 5.7  (n=35) | 17.5 ± 5.7  (n=77) |
|  | Change | 0.5 ± 3.5 | 1.0 ± 3.1 | 0.7 ± 3.3 |
| General psychopathology scale | Baseline | 29.1 ± 6.1  (n=82) | 27.5 ± 6.2  (n=79) | 28.3 ± 6.2  (n=161) |
|  | Week 48 | 30.0 ± 8.2  (n=42) | 28.4 ± 8.2  (n=35) | 29.3 ± 8.2  (n=77) |
|  | Change | 0.9 ± 8.0 | 0.9 ± 7.0 | 0.9 ± 7.5 |
| Composite scale | Baseline | −4.9 ± 5.1  (n=82) | −4.7 ± 5.6  (n=79) | −4.8 ± 5.3  (n=161) |
|  | Week 48 | −5.5 ± 6.1  (n=42) | −6.4 ± 4.6  (n=35) | −5.9 ± 5.5  (n=77) |
|  | Change | −0.3 ± 3.3 | −1.1 ± 3.2 | −0.7 ± 3.3 |

Data are mean ± standard deviation.

JCDSS, Calgary Depression Scale for Schizophrenia, Japanese version; MADRS-J, Montgomery–Asberg Depression Rating Scale, Japanese version; PANSS, positive and negative symptom scale; YMRS, Young Mania Rating Scale
